# Influenza Pandemic Vaccines: Spread Them Thin?

**DOI:** 10.1371/journal.pmed.0040228

**Published:** 2007-06-19

**Authors:** Christophe Fraser

## Abstract

Fraser discusses a new study that uses exploratory modeling to tackle the difficult issue of what to do with limited stockpiles of pre-prepared influenza pandemic vaccines.

The global spread of highly pathogenic H5N1 influenza through poultry flocks is driving a large research effort aimed at mitigating the worst effects of an eventual human pandemic. Mathematical models and computer simulations are being used to explore different policy options in influenza pandemic control (for examples, see [1–4]). In a new study published in *PLoS Medicine*, Riley, Wu, and Leung [5] use such exploratory modelling to tackle the thorny issue of what to do with limited stockpiles of pre-prepared influenza pandemic vaccines [5].

## The Study's Key Findings

Several vaccines matched to circulating avian H5N1 strains are in development [6–8], one has already been licensed by the US Food and Drug Administration [8,9], and several more are in the pipeline. Vaccine stockpiles are being planned and amassed. The issue of what to do with these stockpiles has been the subject of intense discussion. Decisions need largely to be taken in advance because of the time needed for the vaccine to be distributed and to take effect after inoculation. Some governments (e.g., the US) have drafted specific plans to prioritize vaccination of those individuals who are crucial to controlling a pandemic (such as front-line medical staff or vaccine producers) or those who are at heightened risk of influenza-related complications (such as pregnant women or the elderly) [10].

Rather than trying to prioritise individuals by their clinical need or role in pandemic response, Riley et al. start instead from the premise that the vaccine may be considered as part of the armoury of tools available for pandemic mitigation [5]. They address the interesting question of how best to spread the vaccine throughout the population to reduce the total number of people infected in an eventual outbreak. They come to the (perhaps surprising) conclusion that, in the case of a limited stockpile, it is best to spread the vaccine thinly; i.e., that a low dose of vaccine given to many people is in many cases more effective than a large dose given to few. This approach is also known as antigen sparing (see Glossary).

**Linked Research Article**

This Perspective discusses the following new study published in *PLoS Medicine:*

Riley S, Wu TJ, Leung GM (2007) Optimizing the dose of pre-pandemic influenza vaccines to reduce the infection attack rate. PLoS Med 4: e218. doi:10.1371/journal.pmed.0040218

Steven Riley and colleagues examine the potential benefits of “stretching” a limited supply of vaccine and suggest that substantial reductions in the attack rate are possible if vaccines are given to more people at lower doses.

## Models Calibrated to Past Pandemics

The international effort to control or mitigate an eventual pandemic has been substantially boosted since 2004 by the realisation that the three influenza pandemics of the twentieth century (1918, 1957, and 1968) were characterised by relatively low transmissibility, as measured by the basic reproduction number, *R*_0_ [1,2,11–19]. This number is an estimate of how many people a typical case infects over his or her infectious period, and has been estimated to lie between 1.5 and 4 for all three pandemics. There are subtleties in the estimation of *R*_0_, such as a reliance on good estimates of the infection generation time distribution [12], and on aggregated city-wide data (except for [15], in which *R*_0_ is estimated in a military camp outbreak).

It is worth appreciating these subtleties, as so much of what is predicted in simulation scenarios depends on *R*_0_. Analysing records from historical pandemics should continue to be a priority. Figure 1 gives a simple example of how the same intervention could have drastically different effects in epidemics characterised by different *R*_0_.

**Figure 1:**
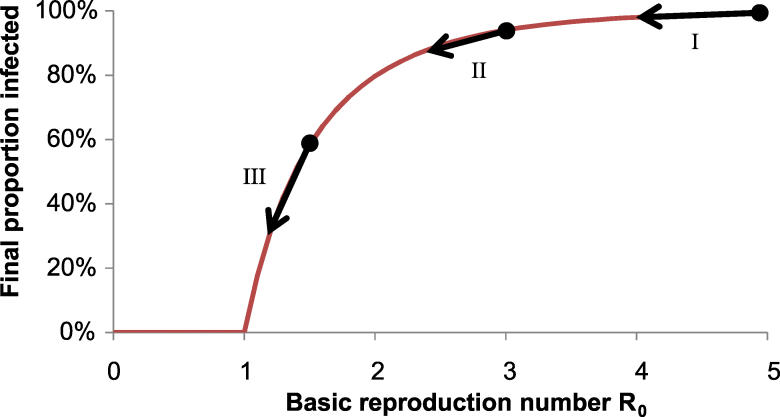
How the Efficacy of Interventions That Limit Transmission Depends on the Basic Reproduction Number, *R*_0_ The final proportion infected in a pandemic for a general class of models with homogeneous population mixing is plotted as a function of *R*_0_ [22,23]. The arrows highlight the effect of an intervention that reduces transmission by 20% for three different baseline values of *R*_0_. This curve can be used, for example, to predict the effect of a vaccine that directly protects 20% of individuals on those who are not directly protected. In scenario I, *R*_0_ is 5 and the intervention reduces the attack rate by 1.3%. In scenario II, *R*_0_ is 3 and the reduction is 6.2%. In scenario III, *R*_0_ is 1.5 and the reduction is 26.9%

Key to understanding this dependency is the notion of herd effect: by vaccinating an individual, not only is that person protected, but his or her contacts are also at reduced risk of infection. This herd effect is also seen with antiviral treatment, prophylaxis, social isolation, or indeed any intervention that reduces transmission. Based on estimates of *R*_0_ for past epidemics, this herd effect could be very large. Consider, for example, effectively vaccinating 10% of individuals against an epidemic disease with *R*_0_ = 1.5. Figure 1 predicts that the final attack rate (i.e., the total number who get influenza) amongst those not protected by the vaccine would be reduced from 58% to 47%; i.e., in this case there would be nearly as many people protected indirectly by the herd effect as directly by the vaccine. One proposal aimed at maximizing the herd effect for influenza is to prioritize vaccination of children (who are the most infectious group) over the elderly (who are among those most at risk of complications) [20], an approach considered by Riley et al. [5] (with mixed conclusions).

A fundamental issue with attempting to maximize the herd effect is that whereas the direct effects can be targeted at individuals, the herd effect protects more randomly. Some people may be uncomfortable with approaches, such as those suggested by Riley et al. [5], that trade losses in direct vaccine efficacy in those targeted for collective gains in overall reduced attack rate.

## Limitations of the Study and Challenges for Future Research

Riley and colleagues' results are quite dependent on the assumed nature of immunity, and they consider a number of different possible immunological assumptions. In the first case they assume that for any given dose, a proportion of vaccine recipients are fully protected and the rest not at all (an all-or-nothing response). In this case it is possible to explain the study results quite simply. The number protected is made greater by vaccinating more people with a lower dose. This conclusion is, of course, heavily dependent on the relation between antibody titre and immune response; for example, if there was a threshold below which a dose gave no protection at all, there would be no sense in vaccinating below that titre.

If, on the other hand, vaccination induces more homogenous partial protection, the situation is more complex. The herd effect needs to be fully accounted for and is strongly dependent on *R*_0_, which they take to be 1.8. Some may take comfort from the consistency of estimates of *R*_0_ for the past three pandemics, but the dependence of Riley and colleagues' results on their assumed value of *R*_0_ is not the only issue here. The circulating virus will not be the currently known avian H5N1, but rather a newly human-adapted strain, and the match between vaccines based on an avian virus and one based on a human-adapted pandemic strain are unknown. The authors address the dependence of their model on the potential mismatch partially, and the data needed to characterise this match could be collected quickly when a new virus is identified.

**Glossary**

**Antigen sparing:** Reducing the dose administered in order to vaccinate more people. A general concept that includes dose-reducing strategies such as the one considered here, as well as use of an adjuvant or alternative vaccine delivery methods to boost the immune response for a given dose.

***R*_0_:** Basic reproduction number, the number of people infected by a typical case during an exponentially growing epidemic. A key measure of transmissibility.

**Generation time distribution:** The distribution of time intervals between being infected and infecting others.

**Herd effect:** The indirect effect of interventions against infectious diseases in which people not targeted by the interventions are protected by breaks in the chains of transmission.

**Epidemiological synergy:** The phenomenon in which combined interventions to reduce infectious disease transmission achieve more than the sum of the effect of each intervention implemented alone. Linked to the herd effect.

In the most pessimistic scenario, the available vaccines might provide only partial protection even with high doses, and any dilution would further reduce individual protection with little or no compensation from increased herd immunity.

A fundamental problem the authors had was that, while data on induced haemagglutinin titres are widely available for candidate vaccines, the relation between these titres and actual protection has been poorly studied. Riley et al. resorted to using some human challenge studies from the 1970s to calibrate their models, but these studies have their limitations. There is no guarantee that the relation between haemagglutinin titre and protection is universal.

## What to Do With the Results?

Mathematical models and computer simulations are not crystal balls, but rather tools that allow questions to be precisely defined, assumptions to be made clear, and logical deductions to be performed. So construed, they can contribute a great deal to the policy debate.

Thus, in this light, this study offers some important points of discussion and action. The numbers provided by Riley et al. should perhaps not be seen as predictions, but rather examples based on extrapolating from past pandemics used to illustrate the potential for antigen sparing. The magnitude of reduction in attack rate that the authors discuss in their models is quite modest (from 0% to 20%), but this still represents many infections prevented. However—and here the authors may have been overly conservative—when added to the other interventions that would likely be carried out, the potential herd effect would grow, and the reductions in attack rate would be greater because of epidemiological synergy.

More work clearly needs to go into establishing the quantitative correlates of immunity for influenza, which cannot easily be done for pandemic virus strains. Nevertheless, good data for different strains of seasonal influenza could go a long way. Not only do we need cross-immunity tables for vaccines and virus strains, but also studies that correlate different measures of the immune response to actual protection from infection. These data could be derived from challenge experiments or household secondary attack rate studies; the latter could also be used to determine potential effects of vaccination on infectiousness, as has been done for antiviral drugs [21]. Such data would help to make more sense of the studies of cross-reactivity between vaccine and pandemic strain that would inevitably be carried out in the first few weeks of an emerging pandemic. The data would allow these and other related analyses to be rapidly revised as necessary.

Most importantly, some reflection is needed on the real aims of pre-pandemic vaccination: is the aim to minimise the overall attack rate or to protect specific individuals or groups? We must also consider whether anyone is ready for the potential consequences of deploying a suboptimal vaccine in an uncertain attempt to maximise our herd protection, with a possible reduction in the extent of protection of individuals.

Some may feel that publishing this work about vaccines with unknown antigenicity elicited against an unknown virus is jumping the gun. The most important aspect of Riley et al.'s paper is that it brings forward a new, interesting concept for wider discussion in the public domain.

## References

[pmed-0040228-b001] Ferguson NM, Cummings DA, Cauchemez S, Fraser C, Riley S (2005). Strategies for containing an emerging influenza pandemic in Southeast Asia. Nature.

[pmed-0040228-b002] Ferguson NM, Cummings DA, Fraser C, Cajka JC, Cooley PC (2006). Strategies for mitigating an influenza pandemic. Nature.

[pmed-0040228-b003] Germann TC, Kadau K, Longini IM, Macken CA (2006). Mitigation strategies for pandemic influenza in the United States. Proc Natl Acad Sci U S A.

[pmed-0040228-b004] Longini IM, Nizam A, Xu S, Ungchusak K, Hanshaoworakul W (2005). Containing pandemic influenza at the source. Science.

[pmed-0040228-b005] Riley S, Wu JT, Leung GM (2007). Optimizing the dose of pre-pandemic influenza vaccines to reduce the infection attack rate. PLoS Med.

[pmed-0040228-b006] Bresson JL, Perronne C, Launay O, Gerdil C, Saville M (2006). Safety and immunogenicity of an inactivated split-virion influenza A/Vietnam/1194/2004 (H5N1) vaccine: Phase I randomised trial. Lancet.

[pmed-0040228-b007] Lin J, Zhang J, Dong X, Fang H, Chen J (2006). Safety and immunogenicity of an inactivated adjuvanted whole-virion influenza A (H5N1) vaccine: A phase I randomised controlled trial. Lancet.

[pmed-0040228-b008] Treanor JJ, Campbell JD, Zangwill KM, Rowe T, Wolff M (2006). Safety and immunogenicity of an inactivated subvirion influenza A (H5N1) vaccine. N Engl J Med.

[pmed-0040228-b009] (2007 April 17). FDA approves First U.S. vaccine for humans against the avian influenza virus H5N1. http://www.fda.gov/bbs/topics/NEWS/2007/NEW01611.html.

[pmed-0040228-b010] US Department of Health and Human Services (2007). HHS pandemic influenza plan, Appendix D. http://www.hhs.gov/pandemicflu/plan/appendixd.html.

[pmed-0040228-b011] Mills CE, Robins JM, Lipsitch M (2004). Transmissibility of 1918 pandemic influenza. Nature.

[pmed-0040228-b012] Wallinga J, Lipsitch M (2007). How generation intervals shape the relationship between growth rates and reproductive numbers. Proc R Soc B Biol Sci.

[pmed-0040228-b013] Chowell G, Ammon CE, Hengartner NW, Hyman JM (2006). Estimation of the reproductive number of the Spanish flu epidemic in Geneva, Switzerland. Vaccine.

[pmed-0040228-b014] Hall IM, Gani R, Hughes HE, Leach S (2007). Real-time epidemic forecasting for pandemic influenza. Epidemiol Infect.

[pmed-0040228-b015] Sertsou G, Wilson N, Baker M, Nelson P, Roberts MG (2006). Key transmission parameters of an institutional outbreak during the 1918 influenza pandemic estimated by mathematical modelling. Theor Biol Med Model.

[pmed-0040228-b016] Massad E, Burattini MN, Coutinho FA, Lopez LF (2007). The 1918 influenza A epidemic in the city of Sao Paulo, Brazil. Med Hypotheses.

[pmed-0040228-b017] Gani R, Hughes H, Fleming D, Griffin T, Medlock J (2005). Potential impact of antiviral drug use during influenza pandemic. Emerg Infect Dis.

[pmed-0040228-b018] Longini IM, Halloran ME, Nizam A, Yang Y (2004). Containing pandemic influenza with antiviral agents. Am J Epidemiol.

[pmed-0040228-b029] Viboud C, Grais RF, Lafont BA, Miller MA, Simonsen L (2005). Multinational impact of the 1968 Hong Kong influenza pandemic: Evidence for a smoldering pandemic. J Infect Dis.

[pmed-0040228-b020] Longini IM, Halloran ME (2005). Strategy for distribution of influenza vaccine to high-risk groups and children. Am J Epidemiol.

[pmed-0040228-b021] Halloran ME, Hayden FG, Yang Y, Longini IM, Monto AS (2007). Antiviral effects on influenza viral transmission and pathogenicity: observations from household-based trials. Am J Epidemiol.

[pmed-0040228-b022] Kermack WO, McKendrick AG (1927). A contribution to the mathematical theory of epidemics. Proc Royal Soc Lond A.

[pmed-0040228-b023] Ma J, Earn DJ (2006). Generality of the final size formula for an epidemic of a newly invading infectious disease. Bull Math Biol.

